# Facile Fabrication of a Superhydrophobic Surface with Robust Micro-/Nanoscale Hierarchical Structures on Titanium Substrate

**DOI:** 10.3390/nano10081509

**Published:** 2020-07-31

**Authors:** Shuliang Dong, Zhenlong Wang, Libao An, Yaogang Li, Baozhong Wang, Hongchao Ji, Han Wang

**Affiliations:** 1 College of Mechanical Engineering, North China University of Science and Technology, No. 21 Bohai Road, Caofeidian Xincheng, Tangshan 063210, China; lan@ncst.edu.cn (L.A.); jxlyg@ncst.edu.cn (Y.L.); wbzhong@ncst.edu.cn (B.W.); jihongchao@ncst.edu.cn (H.J.); 2 School of Mechatronics Engineering, Harbin Institute of Technology, Harbin 150001, China; wanghan5447@163.com

**Keywords:** superhydrophobic surface, robust micro-/nanoscale hierarchical structures, electrical discharge machining, electrochemical etching, titanium

## Abstract

A superhydrophobic surface with robust structures on a metallic surface could improve its application in various harsh conditions. Herein, we developed a new strategy to fabricate robust micro-/nanoscale hierarchical structures with electrical discharge machining and electrochemical etching on a titanium substrate. After modification by fluorinated silane, the static water contact angle and slide angle of the surface could reach 162 ± 2° and 4 ± 1°, respectively. The superhydrophobic surfaces showed good corrosion resistance and mechanical stability after scratching with sandpapers. In addition, the superhydrophobic surfaces had good self-cleaning performance even in an acidic environment as well as the potential to be used as guiding tracks in droplet microfluidics and lab-on-a-chip systems. These results are expected to be helpful in designing the surface of liquid float gyroscope parts.

## 1. Introduction

Superhydrophobic surfaces with extreme states of surface wettability have received increased attention in many fields [1] such as corrosion resistance [2,3,4], self-cleaning [5,6], and drag reduction [7,8]. A surface is considered superhydrophobic if a water static contact angle (CA) is larger than 150° and a sliding angle (SA) is smaller than 10° [9]. The surface roughness and energy codetermine the superhydrophobic properties [10]. In general, two alternative ways, enhanced surface roughness of low surface energy materials or reduced surface energy of the roughened surface, are popular in fabricating superhydrophobic surfaces [11,12,13,14]. Due to fact that metals do not have the property of low surface energy, a feasible way to obtain superhydrophobic surface on metal is through building a roughened surface and coating with low surface energy materials.

Titanium and its alloys are widely used in the aerospace field for their low density, non–magnetic, and corrosion resistance [15,16]. However, under some harsh conditions, the corrosion resistance behaviors are also expected to be enhanced [17,18]. Research shows that corrosion resistance could be improved by creating a superhydrophobic surface on a material [1,2,3,4]. Up to now, a great number of methods have been used to fabricate superhydrophobicity on titanium and its alloys surfaces. Gao et al. used anodic oxidation with low surface energy modification to fabricate superhydrophobic surfaces on Ti–6Al–4V substrates [19]. Ou et al. prepared superhydrophobic surfaces by 1H,1H,2H,2H-perfluorooctyltrichlorosilane adsorbed onto the etched Ti–6Al–4V alloy [17]. Zhu et al. used a self-assembling method to fabricate a superhydrophobic film on a titanium substrate [20]. Lu et al. developed an electrochemical method to obtain superhydrophobic surfaces by building microstructures in a neutral electrolyte, together with surface energy reduced by a fluoropolymer [21]. Dong et al. fabricated TiO_2_ hierarchical nanostructures on Ti foil by electrochemical anodization. The TiO_2_ hierarchical nanostructures showed superhydrophobic properties after being modified with organic molecules [22]. Although these methods realized superhydrophobic surfaces on titanium or its alloys, these superhydrophobic surfaces are unsuitable for industrial applications due to poor mechanical resistance and a complicated process. 

Electrical discharge machining (EDM) is a non-contact machining technology as well as an effective method for difficult-to-cut material [23]. Furthermore, the surface morphology of an EDM machined surface is beneficial for establishing a superhydrophobic surface [24]. William et al. found that the steel surface was a superhydrophobic surface, then machined micro-mushroom re-entrant structures using micro wire EDM [25]. Bae et al. realized superhydrophobic surfaces on aluminum alloys [26] and stainless steel [27] by machining special microscale grooves with wire EDM. The results showed that the groove direction led to nonuniform wettability with anisotropic properties [26,27]. Wan et al. also found two-direction anisotropy of the superhydrophobic property on steel surfaces with V-shaped grooves [28]. Wan et al. used high speed wire EDM and chemical etching to fabricate micro/nanometer-scale rough structures on an Al alloy surface [29]. The surface could realize the super-amphiphobic property after twice being electric machined, chemically etched, and modified with fluoroalkylsilane. Although these methods provide new ideas for the fabrication of superhydrophobic surfaces, they have limited abilities on curved surfaces. During the EDM process, uniformly distributed microscale cracks appear when the local residual thermal stress in the surface exceeds the material’s ultimate tensile strength [23]. The cracks on the surface are normally regarded as defects degrading anti-corrosion [30]. Undoubtedly, such uniform cracks would lead to the corresponding nanostructures by electrochemical etching (ECE) under the edge effect on the edges of those cracks [31].

In this paper, we present a new strategy to fabricate superhydrophobic surfaces with robust micro-/nanoscale hierarchical structures, which takes full advantage of the micro-cracks formed by EDM on a titanium substrate. The superhydrophobic surface is implemented with the combination of EDM, ECE, and modification by fluorinated silane (1H,1H,2H,2H-perfluorooctadecyltrichlorosilane, FAS). The CA and SA were used to verify the superhydrophobic properties of the surface. The superiority of the superhydrophobic surfaces fabricated by this strategy was proven by corrosion resistance, mechanical abrasion, and self-cleaning tests. 

## 2. Materials and Methods 

### 2.1. Fabrication of Superhydrophobic Surface

In the experiments, titanium (99.99% purity, Her Bei Sai Wei Metallic Material Co. Ltd., Shijiazhuang, China) sheets were cut into a size of 20 mm × 10 mm × 1 mm. Before the EDM process, the workpieces were ultrasonically cleaned successively in ethanol and deionized water. A bulk of graphite was used as the tool electrode with a size of 200 mm × 30 mm × 30 mm. The EDM process was immersed in kerosene (dielectric work solution, EDM-3, Mobil, Irving, TX., USA) on a commercial EDM machine (DR30a, Dimon CNC Technology Co. Ltd., Beijing, China). The discharge current and discharge voltage were 2A and 250 V, respectively. A lay of 0.2 mm was removed from the workpiece to obtain the surface with microscale craters and cracks. The EDM machined surface was cleaned ultrasonically in ethanol. ECE was carried out by DC voltage (IT6302, ITECH Electronic Co. Ltd., Nanjing, China). The EDM machined surface (anode) was faced parallel to the graphite (cathode) sheet with a distance of 25 mm in an electrolyte of 0.1 M hydrochloric acid solution. The process was carried out under a voltage of 10 V with the times of 10 s, 20 s, and 30 s. After ECE, the workpiece was ultrasonically cleaned successively in deionized water and ethanol. Finally, the workpiece was immersed into a 0.5 wt% ethanol solution of FAS for 1 h, then dried it at 120 °C in air for 1 h.

### 2.2. Characterization

A scanning electron microscope (SEM, Supra 55 Sapphire, Carl Zeiss, Oberkochen, Germany) and atomic force microscope (AFM, Dimension Icon, Goettingen, Germany) were used to acquire the morphology of the workpiece. The XRD patterns of the surface of the workpiece were analyzed by x-ray diffractometer (XRD, Empyrean, PANalytical, Almelo, Netherlands) with Cu Kα1, λ = 0.154 nm. Chemical states of elements were analyzed using x-ray photoelectron spectroscopy (XPS, ESCALAB 250XI, Thermo Fisher Scientific, Waltham, MA, USA). The values of CA and SA were tested with a 5 μL droplet of water on an optical contact angle meter system (JC2000C1, Shanghai Zhongchen Digital Technic Apparatus Co. Ltd., Shanghai, China). The average values of CA and SA were obtained from five measurements at different areas on the surface of the workpiece. The Young−Laplace method was used to calculate the static contact angle [32,33]. 

### 2.3. Corrosion and Mechanical Durability Tests of Superhydrophobic Surfaces

The corrosion resistance behavior of the superhydrophobic surface was evaluated by potentiodynamic polarization (Tafel) curves and electrochemical impedance spectroscopy (EIS). The tests were examined in a three-electrode cell with the electrochemical workstation (CHI660D, CH Instruments Inc., Austin, TX, USA) in 3.5 wt% NaCl solution at room temperature. The graphite electrode, workpiece, and silver/silver chloride (Ag/AgCl, 3 M KCl) electrode were the counter electrode, working electrode, and reference electrode, respectively. Tafel curves were carried out at a scanning rate of 1 mV s^−1^. EIS tests were carried out under 10 mV of the amplitude of the perturbation voltage and frequencies ranging from 10^5^ to 10^−1^ Hz. The mechanical durability of the superhydrophobic surface was examined by sandpaper abrasion [34,35]. The superhydrophobic surface moved on sandpaper (Grit No. 1000) with a speed of 18–25 mm s^−1^ and normal pressure of 4.9 kPa and 15 kPa on it. The values of CA and SA were recorded after each 30 cm abrasion. Each test was repeated more than three times to verify the repeatability of the results.

## 3. Results and Discussion

### 3.1. Surface Morphology and Wettability

**Figure 1:**
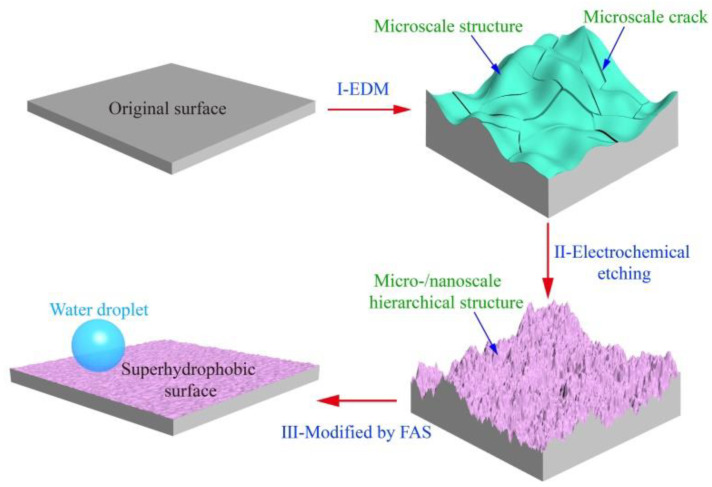
Schematic illustration of the superhydrophobic surface with robust micro-/nanoscale hierarchical structures fabricated on titanium. Step I: Workpiece is machined by the electrical discharge machining (EDM) process, where microscale craters and cracks are generated; Step II: Electrochemical etching (ECE) is used to manufacture nanoscale structures on the EDM machined surface. Step III: The surface with micro-/nanoscale hierarchical structures is modified by 1H,1H,2H,2H-perfluorooctadecyltrichlorosilane (FAS) and dried.

**Figure 2:**
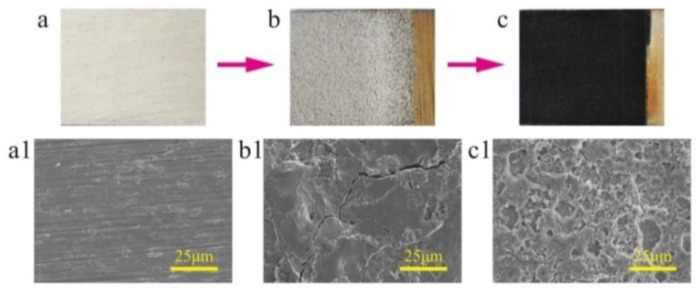
Surface morphology of the titanium surface at each step. (**a**) Original surface, (**b**) EDM surface, (**c**) ECE surface. Scanning electron microscopy (SEM) image of (**a1**–**c1**) is the magnification of (a–c), respectively.

Figure 1 schematically illustrates the strategy for the fabrication of a superhydrophobic surface with robust micro-/nanoscale hierarchical structures on a titanium substrate. This includes three steps. First, the microscale structures on the surface of the workpiece were created by EDM. During the EDM, the spark discharge between the workpiece and tool electrode can melt and vaporize the titanium by local heating form the electric energy, leaving large amounts of uniformly distributed microscale craters and cracks on the surface (Figure 2b,b1). The surface with microscale craters was used as a platform for the superhydrophobic surface. The uniform distribution of microscale cracks is beneficial to the speed and uniformity of ECE because of the edge effect of the microscale cracks [31,36]. Second, the surface was then machined by ECE to form nanoscale structures. In the process of ECE, the nanoscale structures first formed at the edge of the cracks, with the micro-/nanoscale hierarchical structures forming on the titanium substrate (Figure 2c,c1). Meanwhile, the color of the sample surface changed from silvery-white to a dark black (Figure 2c). Third, the surface with micro-/nanoscale hierarchical structures was modified by FAS and dried. After being modified by FAS, the as-prepared titanium surface showed good superhydrophobic properties with CA a value of 162 ± 2° and SA value of 4 ± 1° (advancing/receding angle 164 ± 1°/160 ± 2°).

**Figure 3:**
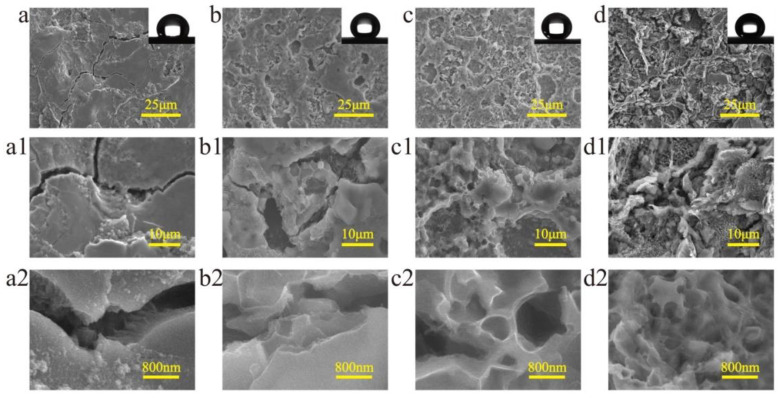
SEM images of the surface morphology of titanium machined by EDM and under different ECE times of (**a**) 0 s (**b**) 10 s, (**c**) 20 s, and (**d**) 30 s. (**a1**–**d2**) is the magnification of (a–d), respectively.

Surface morphology is one of the main factors affecting the superhydrophobic property. In this experiment, surface morphology was mainly determined by ECE time. Figure 3 shows the morphology evolution of the surface machined by EDM and ECE. Figure 3a shows the surface only machined by EDM without ECE, which had a typical surface morphology of the EDM machined titanium surface with craters and cracks. Figure 3a1,a2 shows the enlarged SEM image of the cracks on the surface. The surfaces machined under different ECE times were prepared. Figure 3b–d shows the change in the surface morphology under different ECE times. It can be seen that nanoscale structures first formed at the edge of the crack (Figure 3b,b1,b2). When the ECE time was 20 s, a layer of nanoscale structures covered the EDM machined surface (Figure 3c,c1). However, as the ECE time increased, the microscale craters gradually disappeared with material removal (Figure 3d,d1). 

**Figure 4:**
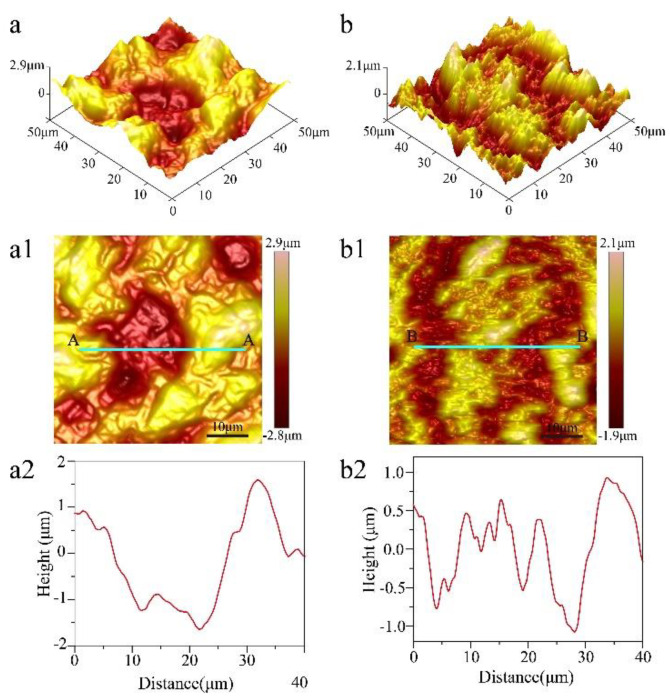
Atomic force microscope (AFM) image surface morphology of titanium machined only by EDM. (**a**) 3D height map, (**a1**) surface height map, and (**a2**) height profile through section A–A for scanned location (**a1**), and machined by EDM and ECE time 20 s. (**b**) 3D height map, (**b1**) surface height map, and (**b2**) height profile through section B–B for the scanned location (**b1**).

In order to analyze the change in the morphologies of titanium at different steps, AFM was used to characterize the surface only machined by EDM and machined by EDM and ECM with 20 s in Figure 4. The 3D height map and surface height map of the titanium surface only machined by EDM are shown in Figure 4a,a1, respectively. It can be seen that the surface was mainly composed of microscale structures and lacked nanoscale structures, which was reinforced through the height profile through section A–A in Figure 4c. Figure 4b shows the 3D height map of the titanium surface machined by EDM and ECM with 20 s, where it can be seen that smaller structures formed on the microscale structures. The height profile through section B–B (Figure 4b2) showed that these smaller structures reached the nanoscale, which indicates the surface had micro-/nanoscale hierarchical structures.

**Figure 5:**
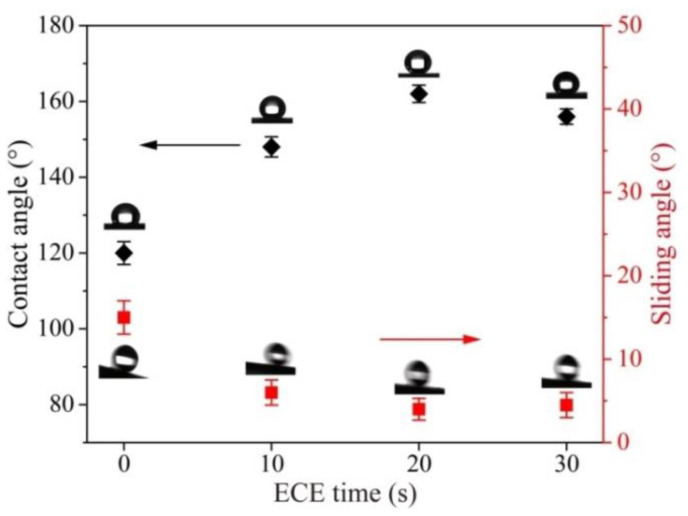
Static water contact angle and sliding angle on EDM surfaces under different ECE times.

In order to test the effect of micro-/nanoscale hierarchical structures on the preparation of the superhydrophobic surface, the original titanium surface modified by FAS was used as a reference. The CA and AS value of the original titanium surface modified by FAS were 95 ± 3° and 46 ± 4° (advancing/receding angle 117 ± 5°/71 ± 4°), respectively. To further investigate the effect of ECE time on superhydrophobic property, the surfaces under different ECE times were modified by FAS under the same configuration. The CA and AS values recorded at ECE times from 0 to 30 s are shown in Figure 5. The ECE time 0 indicates the surface only machined by EDM. The CA increased first and then decreased with the increase in ECE time. Nanoscale structures increased with the arising ECE time (Figure 3b,c), and enhanced the synergistic effect of micro-/nanoscale hierarchical structures. The best superhydrophobic property was evidenced with the CA of 162 ± 2° and SA of 4 ± 1° (advancing/receding angle 164 ± 1°/160 ± 2°), indicating a strong synergistic effect of micro-/nanoscale hierarchical structures at the etching time of 20 s. The as-prepared superhydrophobic surface also showed an ideal resistance to water droplet impingement (Video S1). When the ECE time flies, the CA drops to 156 ± 2°. SA has the opposite tendency compared with the CA as shown in Figure 5. The reason for this is that the microscale structures gradually disappeared as the synergistic effect of the micro-/nanoscale hierarchical structures was reduced (Figure 3d).

**Figure 6:**
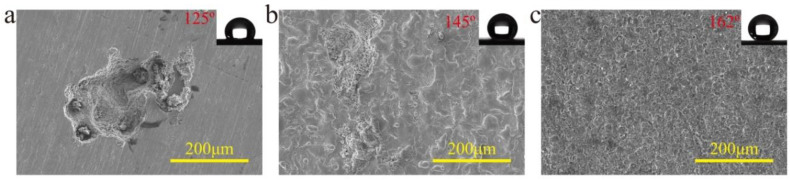
SEM images of surface morphology of different surfaces after ECE. (**a**) Original surface, (**b**) EDM surface without cracks, (**c**) EDM surface with cracks.

To identify the functionality of the cracks on the titanium surface machined by EDM, an original surface and EDM machined surface without cracks were machined by ECE, respectively. During the ECE, both of the surfaces machined with the same parameters compared to that of the EDM machined surface with cracks. Figure 6 shows the SEM images of the surface morphology of different surfaces after ECE. The ECE on the original and EDM machined surface without cracks was nonuniform. After the same surface modification, the CA and SA on the smooth surface were only 125 ± 5° and 23 ± 8° (advancing/receding angle 138 ± 8°/115 ± 7°), as shown in the Figure 6a inset, whereas they were 145 ± 3° and 16 ± 6° (advancing/receding angle 153 ± 6°/137 ± 6°) on the EDM machined surface without cracks, as shown in the Figure 6b inset. Obviously, the surfaces without cracks exhibited a worse superhydrophobicity. Therefore, the EDM machined surface with microscale craters and cracks provided a good basis for ECE to fabricate a superhydrophobic surface.

**Figure 7:**
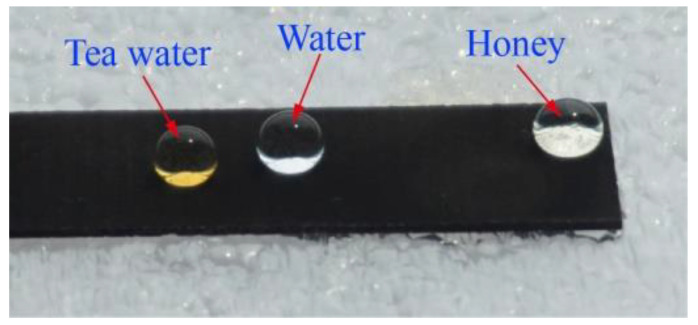
Photograph of different liquid droplets on the as-prepared superhydrophobic surface.

Figure 7 shows the photograph of different liquid droplets on the as-prepared superhydrophobic surface. It can be seen that not only the water droplet, but also the tea water and honey remained in a spherical state on the as-prepared superhydrophobic surface. The CA of the tea water and honey could reach162° and 156°, respectively. All of these easily rolled off the as-prepared superhydrophobic surface under an external force. 

The surface robustness of superhydrophobic coatings is the main and decisive factor of industrial applications because the chemical modification and nanoscale structures are frequently readily damaged [37,38]. Water droplets with a diameter of 2.6 ± 0.3 mm and a velocity of 1.4 m s^−1^ were used to simulate the rain in real nature. During the experiment, water droplets dropped continuously onto the as-prepared superhydrophobic surface and the results showed that the water repellency ability of the as-prepared superhydrophobic surface did not cause apparent damage for at least five days. This showed a significantly improved durability when compared with the sprayed TiO_2_ nanoparticle [39].

### 3.2. Chemical Composition

**Figure 8:**
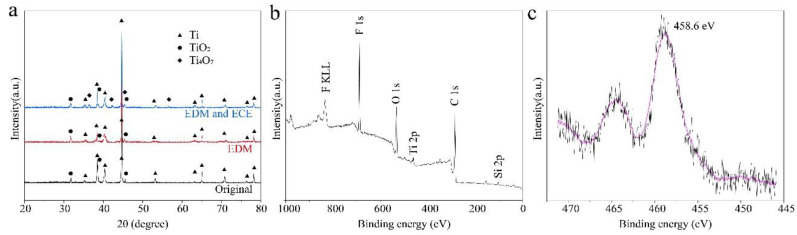
Characterization of the chemical structure of surfaces under different processes. (**a**) XRD pattern of the original titanium surface, EDM surface, and EDM and ECE process, (**b**) XPS of the survey spectrum of the as-prepared superhydrophobic surface, (**c**) high-resolution XPS scanning of the Ti 2p spectrum of the as-prepared superhydrophobic surface.

The XRD patterns of the titanium surface under different processes were used to verify the chemistry changes. Figure 8 shows the XRD patterns of the original surface, EDM machined surface, and EDM and ECE machined surface. The XRD patterns of the sample before and after EDM had diffraction Ti (ICSD 97-003-9166) peaks and TiO_2_ (ICSD 97-003-5124) peaks. The oxidation occurred because of the exposure of titanium to the ambient environment [19]. Although the EDM process was immersed in the kerosene, oxidation of the titanium surface was inevitable because the instantaneous temperature was over the range 8000–12,000 °C on titanium surfaces [40]. After being machined by ECE, the Ti_4_O_7_ (JCPDF #50-0787) phase appeared, which explains the color change of the titanium surface from silvery-white to a dark black (Figure 2c). XPS was carried out to reveal the chemical component of the as-prepared superhydrophobic surface. It can be seen from the survey spectrum (Figure 8b) that a strong fluorine peak was located at 688 eV, and peaks of O, C, and Si were observed, which demonstrated that the micro-/nanoscale hierarchical structures were effectively covered by the FAS, and surface energy was greatly reduced [41]. Figure 8c shows the high-resolution XPS scanning of Ti 2p spectrum of the as-prepared superhydrophobic surface. The peak of Ti 2p shifted positively to 458.6 eV, which indicated that the FAS molecules were chemically anchored onto the micro-/nanoscale hierarchical structures via “Ti–O–Si” bonds [17,42,43].

### 3.3. Corrosion Resistance

**Figure 9:**
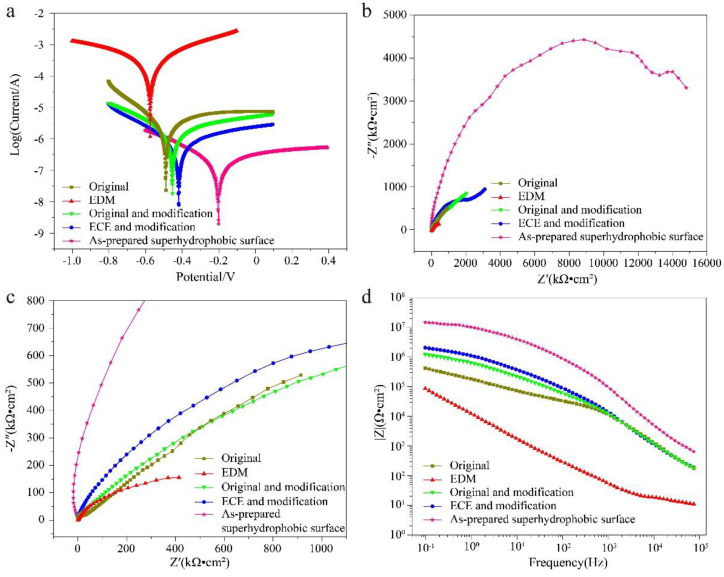
Corrosion resistance of the original titanium surface, EDM surface, original and modification, ECE and modification surface, and the as-prepared superhydrophobic surface in neutral solution (3.5 wt% NaCl) for 30 min. (**a**) Tafel curves, (**b**) Nyquist plots, (**c**) Enlarged scope of (**b**) Nyquist plots in the high frequency range, (**d**) Bode plots.

Corrosion resistance performance has an important effect on the service life of superhydrophobic surfaces [44]. The corrosion resistance of the titanium surface after different processes was tested. The results were revealed by the Tafel curves and EIS. The Tefel curves of the titanium surface at different processes are shown in Figure 9a. Table 1 shows the details of the *E_corr_* and *i_corr_* acquired from the Tafel curves by Tafel extrapolation [45,46]. The *E_corr_* of the as-prepared superhydrophobic surface could reach up to −0.20 V, which is quite a bit more positive than that of the original surface (−0.48 V), EDM surface (−0.58 V), original and modification (−0.45 V), and ECE and modification surface (−0.42 V), indicating the as-prepared superhydrophobic surface with the best corrosion resistance. The *i_corr_* of the as-prepared superhydrophobic surface reached 5.01 × 10^−8^ A cm^−2^, which is three orders of magnitude lower than the EDM surface (7.94 × 10^−5^ A cm^−2^), two orders of magnitude lower than the original surface (1.26 × 10^−6^ A cm^−2^), and one order of magnitude lower than the original and modification (5.62 × 10^−7^ A cm^−2^), and ECE and modification surface (2.51 × 10^−7^ A cm^−2^), indicating the as-prepared superhydrophobic surface with the lowest corrosion rate [47,48,49]. The *E_corr_* of the EDM surface (−0.58 V) was more negative than that of the original surface (−0.48 V), and the *i_corr_* of the EDM surface (7.94 × 10^−5^ A cm^−2^) was an order of magnitude higher than that of the original surface (1.26 × 10^−6^ A cm^−2^), showing that the corrosion resistance of the titanium surface was reduced by EDM. The reason for this is that many microscale cracks, which promote the occurrence of corrosion [36], are formed on the titanium surface during EDM. The inhibition efficiency (*η*) of the titanium surface at different processes can be calculated by [50,51]:

(1) $$\eta =\frac{i_{0}-i_{corr}}{i_{0}}\times 100\%$$

where *i*_0_ represents the current density of the original titanium surface, and *i_corr_* represents the current density from the other titanium surface. The *η* of the EDM surface was −6210%, also showing that the EDM surface was more prone to corrosion than the original surface. The *η* of the as-prepared superhydrophobic surface was 96.02% (Table 1), which showed that the excellent corrosion resistance ability was excellent.

Furthermore, anticorrosion and electrochemical corrosion behaviors of the titanium surface at different processes were evaluated by EIS tests. The Nyquist plots and Bode plots of the titanium surfaces at different processes are shown in Figure 9b,c. In Nyquist plots, the impedance semicircle diameter is related to polarization resistance, and a larger impedance semicircle diameter represents better corrosion resistance [24,47]. Significantly, the impedance semicircle diameter of the as-prepared superhydrophobic surface increases sharply, approaching ten thousand kΩ·cm^2^ (Figure 9b), about 16 times compared to the original surface.

The impedance value |Z| of the as-prepared superhydrophobic surface reached up to 15,200 kΩ·cm^2^ at 0.1 Hz (Figure 9d). In the low-frequency region, the |Z| of the as-prepared superhydrophobic surface was two orders of magnitude larger than that of the original titanium surface, and one order of magnitude larger than that of the original and modification, the ECE, and modification surface. According to the Nyquist plots and Bode plots, the as-prepared superhydrophobic surface provides excellent corrosion protection for a titanium substrate.

### 3.4. Mechanical Stability

**Figure 10:**
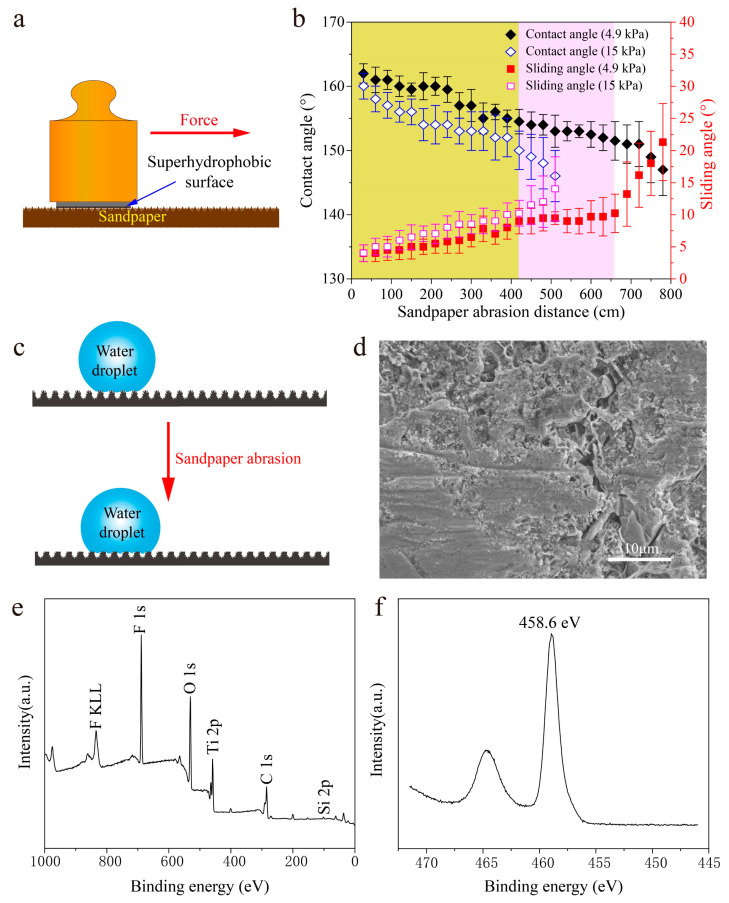
Mechanical stability of the as-prepared superhydrophobic surface. (**a**) Illustration of the abrasion test for the as-prepared superhydrophobic surface. (**b**) Plot of the changes of CA and SA under different mechanical abrasion distances. (**c**) The anti-wear schematic of the as-prepared superhydrophobic surface. (**d**) Surface morphology of the as-prepared superhydrophobic surface after 660 cm abrasion distance. (**e**) XPS of survey spectrum of the as-prepared superhydrophobic surface after 660 cm abrasion distance. (**f**) High-resolution XPS scanning of the Ti 2p spectrum of the as-prepared superhydrophobic surface after a 660 cm abrasion distance.

The poor mechanical stability of most currently developed superhydrophobic surface limits their industrial applications because the surface structures are mechanically weak and can be destroyed even by a slight scratch or abrasive force [52]. The sandpaper abrasion tests were carried out to test the as-prepared superhydrophobic surface mechanical stability. During the sandpaper abrasion tests, the as-prepared superhydrophobic surface was in contact with the sandpaper moving under normal pressure (Figure 10a). After each abrasion of 30 cm, the as-prepared superhydrophobic surface was cleaned by N₂ (0.1 MPa), which removed the impact of dust and sand particles shed from sandpaper on the CA and SA. The CA and SA were measured after each 30 cm and summarized in Figure 10b. Before long abrasion distances of 660 cm, the CA and SA was maintained at 152–162° and 4–10°, respectively. To further test the mechanical stability of the as-prepared superhydrophobic surface, the load on the surface was increased to 15 kPa. It can be seen that the as-prepared superhydrophobic surface lost its superhydrophobic property after an abrasion distance of 420 cm. Meanwhile, the SA was measured to be over 10° (Figure 10b). The results of the sandpaper abrasion tests indicate that the as-prepared superhydrophobic surface had good mechanical abrasion resistance and durability [53,54,55].

The anti-wear schematic of the as-prepared superhydrophobic surface is shown in Figure 10c. During the sandpaper abrasion test, the nanoscale structures on the protrusion areas were damaged by scratching, whereas the nanoscale structures in the craters were protected. Figure 10d shows the surface morphology of the as-prepared superhydrophobic surface after a 660 cm abrasion distance. It can be seen that the micro-/nanoscale hierarchical structures were still on the surface. The microscale structures increased the wear resistance of the surface, which were formed by EDM [56,57]. XPS of the survey spectrum was carried out to confirm the chemical component of the as-prepared superhydrophobic surface after a 660 cm abrasion distance. It can be seen that the peaks of F, O, C, Ti, and Si with the same position can also be found in Figure 10e compared with that in Figure 8b. The peak of the Ti 2p spectrum in Figure 10f shows that the “Ti–O–Si” bonds still existed, which ensured that the as-prepared superhydrophobic surface had good hydrophobic performance even after long distance mechanical abrasion [17,42,43].

### 3.5. Self-Cleaning Property

**Figure 11:**
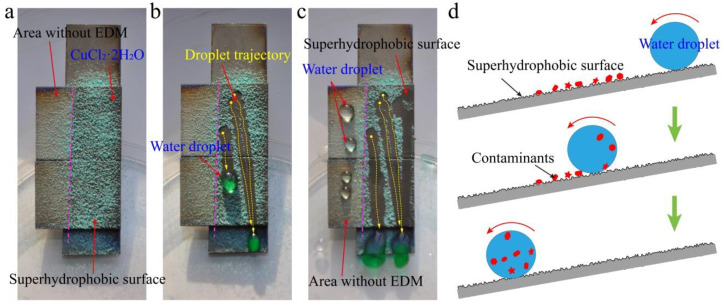
Self-cleaning performance of the as-prepared superhydrophobic surface. (**a**) Artificial dirt (CuCl_2_·2H_2_O) spread on the as-prepared superhydrophobic surface. (**b**) Dirt taken away from the as-prepared superhydrophobic surface by the rolling off of water droplets. (**c**) Comparison of the self-cleaning performance on different surfaces. (**d**) Mechanism of the self-cleaning process of the as-prepared superhydrophobic surface.

The contamination caused by dust can be effectively solved by self-cleaning, which expands the practical application range of superhydrophobic surfaces [1,5]. In order to test self-cleaning performance, an artificial dirt (CuCl_2_·2H_2_O) was spread onto the as-prepared superhydrophobic surface (Figure 11a). When water droplets rolled across the as-prepared superhydrophobic surface, the CuCl_2_·2H_2_O powder was collected and carried away, leaving a cleaning surface along the flow trajectory of water droplets (Figure 11b). Meanwhile, the color of the rolling droplets became green, which indicates that the dirt was successfully removed from the as-prepared superhydrophobic surface. Under the same conditions, water droplets on the surface of the area without EDM were used to compare the self-cleaning performance of the as-prepared superhydrophobic surface. The water droplets on the surface of the area without EDM could not collect the dirt and take it away (Figure 11c and Video S2). Figure 11d shows the mechanism of the dirt picked up by the water droplet from the as-prepared superhydrophobic surface. This test confirmed the self-cleaning performance of the as-prepared superhydrophobic surface. In addition, the solution of copper chloride is an acidic solution, which indicates that the as-prepared superhydrophobic surface has excellent self-cleaning effect under an acidic environment.

### 3.6. Transport of Water Droplets

**Figure 12:**
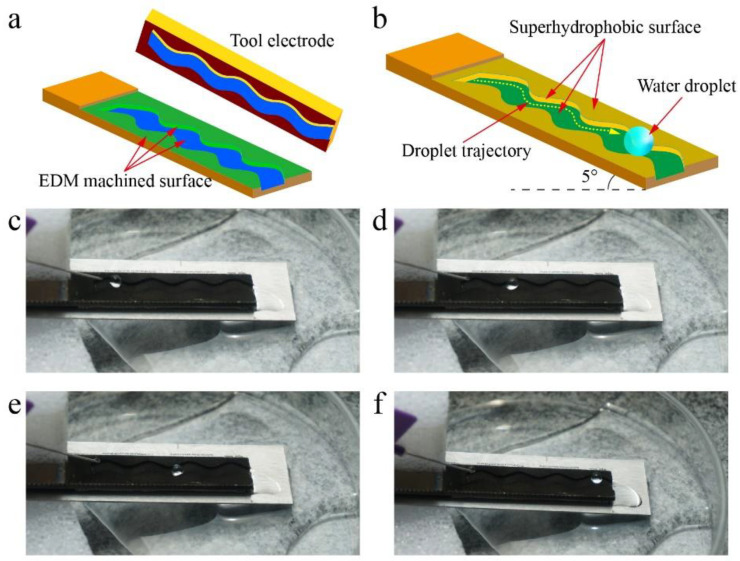
Water droplet transported on the superhydrophobic surface. (**a**) Complex trajectory machined by EDM. (**b**) Mechanism of the water droplet transported on the as-prepared superhydrophobic surface. (**c**–**f**) Photograph of the water droplet moving along the trajectory on the as-prepared superhydrophobic surface.

Water droplets transported on a superhydrophobic surface with low friction could be used in the field of droplet microfluidics and lab-on-a-chip systems [58,59]. The tool electrode with a curved shape (Figure 12a) was used to machine complex trajectory on original titanium surface by EDM. Then, surface superhydrophobicity was realized by the method above-mentioned. Figure 12b shows the mechanism of the water droplet transported on the as-prepared superhydrophobic surface. The as-prepared superhydrophobic surface was fixed on an inclined plane with an inclination angle of 5°, which ensured that the water droplet moved under the action of gravity. 

Figure 12c–f and Video S3 show the water droplet moving along the trajectory on the as-prepared superhydrophobic surface where the water droplet can be seen moving along the designed trajectory. In addition, the water droplet did not stick to the surface, which indicates that the water droplet was not lost during transmission. This experiment shows that the as-prepared superhydrophobic surface has the potential to be used as guiding tracks in droplet microfluidics and lab-on-a-chip systems.

## 4. Conclusions

In this paper, we presented a novel method to fabricate superhydrophobic surfaces with durable and robust micro-/nanoscale hierarchical structures on a titanium surface through the surface topography of EDM. EDM was used to fabricate uniformly distributed microscale craters and cracks on a titanium surface. The EDM surface provided an ideal platform for ECE to form micro-/nanoscale hierarchical structures. On one hand, the uniformly distributed microscale craters provide the base structure for the preparation of the superhydrophobic surface. On the other hand, the uniformly distributed microscale cracks are conducive to fabricating uniform nanostructures by ECE with the edge effect of the microscale cracks. After modification by FAS, the surface with micro-/nanoscale hierarchical structures possessed excellent superhydrophobicity with a CA up to 162 ± 2° and SA low of 4 ± 1° (advancing/receding angle 164 ± 1°/160 ± 2°). The corrosion resistance of the as-prepared superhydrophobic was enhanced 16 times when compared to the original titanium surface and confirmed by electrochemical measurement. The as-prepared superhydrophobic surface showed good durability under the mechanical abrasion test. Furthermore, the as-prepared superhydrophobic surface has the potential to be used as the guiding tracks in droplet microfluidics and lab-on-a-chip systems.

## Figures and Tables

**Table 1:** Details of Tafel polarization curves of titanium samples at different processes in the neutral solution (3.5 wt% NaCl).

Sample	*E_corr_* (V)	*i_corr_* (A cm^−2^)	*η* (%)
Original	−0.48	1.26 × 10^−6^	-
EDM	−0.58	7.94 × 10^−5^	−6201.59
Original and modification	−0.45	5.62 × 10^−7^	55.40
ECE and modification	−0.42	2.51 × 10^−7^	80.08
As-prepared superhydrophobic surface	−0.20	5.01 × 10^−8^	96.02

## Supplementary Materials

The following are available online at https://www.mdpi.com/2079-4991/10/8/1509/s1, Video S1: Water droplet impact on the as-prepared superhydrophobic surface; Video S2: Self-cleaning performance of the as-prepared superhydrophobic surface; Video S3: Liquid transportation.
